# A New Approach to Treating Neurodegenerative Otologic Disorders

**DOI:** 10.1089/biores.2018.0017

**Published:** 2018-07-01

**Authors:** Walter H. Moos, Douglas V. Faller, Ioannis P. Glavas, David N. Harpp, Michael H. Irwin, Iphigenia Kanara, Carl A. Pinkert, Whitney R. Powers, Kosta Steliou, Demetrios G. Vavvas, Krishna Kodukula

**Affiliations:** ^1^ Department of Pharmaceutical Chemistry, School of Pharmacy, University of California San Francisco, San Francisco, California.; ^2^ ShangPharma Innovation, Inc., South San Francisco, California.; ^3^ Department of Medicine, Boston University School of Medicine, Boston, Massachusetts.; ^4^ Cancer Research Center, Boston University School of Medicine, Boston, Massachusetts.; ^5^ Department of Ophthalmology, New York University School of Medicine, New York, New York.; ^6^ Department of Chemistry, Office for Science & Society, McGill University, Montreal, Canada.; ^7^ Department of Pathobiology, College of Veterinary Medicine, Auburn University, Auburn, Alabama.; ^8^ Embassy of Greece in Moscow, Moscow, Russia.; ^9^ Department of Biological Sciences, College of Arts and Sciences, The University of Alabama, Tuscaloosa, Alabama.; ^10^ Department of Health Sciences, Boston University, Boston, Massachusetts.; ^11^ Department of Anatomy, Boston University School of Medicine, Boston, Massachusetts.; ^12^ PhenoMatriX, Inc., Natick, Massachusetts.; ^13^ Retina Service, Angiogenesis Laboratory, Massachusetts Eye and Ear Infirmary, Boston, Massachusetts.; ^14^ Department of Ophthalmology, Harvard Medical School, Boston, Massachusetts.; ^15^ Bridgewater College, Bridgewater, Virginia.

**Keywords:** carnitine esters, epigenetics, hearing loss, lipoic acid, mitochondrial dysfunction, pharmaceutical

## Abstract

Hearing loss, the most common neurological disorder and the fourth leading cause of years lived with disability, can have profound effects on quality of life. The impact of this “invisible disability,” with significant consequences, economic and personal, is most substantial in low- and middle-income countries, where >80% of affected people live. Given the importance of hearing for communication, enjoyment, and safety, with up to 500 million affected globally at a cost of nearly $800 billion/year, research on new approaches toward prevention and treatment is attracting increased attention. The consequences of noise pollution are largely preventable, but irreversible hearing loss can result from aging, disease, or drug side effects. Once damage occurs, treatment relies on hearing aids and cochlear implants. Preventing, delaying, or reducing some degree of hearing loss may be possible by avoiding excessive noise and addressing major contributory factors such as cardiovascular risk. However, given the magnitude of the problem, these interventions alone are unlikely to be sufficient. Recent advances in understanding principal mechanisms that govern hearing function, together with new drug discovery paradigms designed to identify efficacious therapies, bode well for pharmaceutical intervention. This review surveys various causes of loss of auditory function and discusses potential neurological underpinnings, including mitochondrial dysfunction. Mitochondria mitigate cell protection, survival, and function and may succumb to cumulative degradation of energy production and performance; the end result is cell death. Energy-demanding neurons and vestibulocochlear hair cells are vulnerable to mitochondrial dysfunction, and hearing impairment and deafness are characteristic of neurodegenerative mitochondrial disease phenotypes. Beyond acting as cellular powerhouses, mitochondria regulate immune responses to infections, and studies of this phenomenon have aided in identifying nuclear factor kappa B and nuclear factor erythroid 2-related factor 2/antioxidant response element signaling as targets for discovery of otologic drugs, respectively, suppressing or upregulating these pathways. Treatment with free radical scavenging antioxidants is one therapeutic approach, with lipoic acid and corresponding carnitine esters exhibiting improved biodistribution and other features showing promise. These compounds are also histone deacetylase (HDAC) inhibitors, adding epigenetic modulation to the mechanistic milieu through which they act. These data suggest that new drugs targeting mitochondrial dysfunction and modulating epigenetic pathways via HDAC inhibition or other mechanisms hold great promise.

## Background

Vision is the pre-eminent sensory means by which we navigate the world around us,^1^ while the ability to hear endows us with the power of voice communication. It enriches our lives with the sound of music and alerts us to imminent danger that can be heard although perhaps not seen. The Greek physician-philosopher Alcmaeon of Croton^2–5^ and his two protagonist followers, Praxagoras of Kos^3,6^ and the great Alexandrian physician of Chalcedon,^7^ Herophilus,^2,3^ propounded that hearing is a construct of the brain, where external sound channeled to it through the ears (transduced into sensorineural signals) is interpreted.^6^

**FIG. 1.**
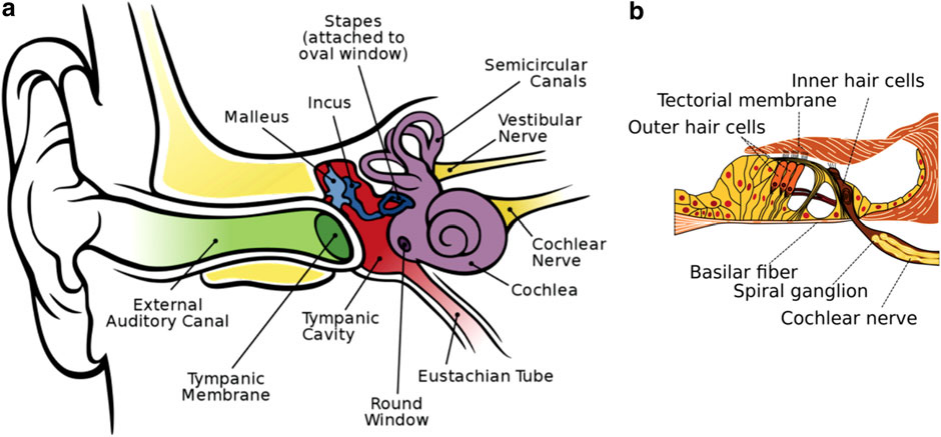
Anatomy of the human ear. **(a)** The outer ear includes the ear lobe and auditory canal; middle ear, the tympanic membrane and cavity; the inner ear, the hearing (cochlea) and balance (vestibular system) organs and the attached associated nerves connecting to the brain. (Figure reproduced from Chittka L, Brockmann A. Perception space—the final frontier. PLoS Biol. 2005;3(4):e137. CC BY 2.5 (https://creativecommons.org/licenses/by/2.5/deed.en), via Wikimedia Commons. Original File URL: https://commons.wikimedia.org/wiki/File:Anatomy_of_the_Human_Ear_en.svg) **(b)** Expanded cross section of the cochlea (organ of Corti) showing the outer and inner hair cells, and the spiral ganglion of the cochlear nerve. (Cochlea-crosssection.png. CC BY-SA 3.0 US (https://creativecommons.org/licenses/by-sa/3.0/us/), via Wikimedia Commons. Original File URL: https://commons.wikimedia.org/wiki/File:Organ_of_corti.svg)

Hearing loss is the most common neurological disorder affecting people worldwide.^8^ The World Health Organization (WHO) estimates that the annual cost of unaddressed hearing loss is in the range of $750–790 billion globally.^9^ In the “Global Burden of Disease,” impaired hearing represents the fourth leading cause of disability worldwide.^10–12^ About 5–7% of the world's population (∼360–500 million people) has a hearing disability^12,13^ that is severe enough in more than 80% of people older than 85 years to interfere with their ability to communicate effectively.^10^ In children, impaired hearing can impinge on their academic potential and social development^13^ with lifelong adverse consequences.^14^ Hearing loss due to mechanical (conductive) and/or anatomical issues in the outer and/or middle ear (Fig. 1) is less prevalent than that resulting from dysfunction in the cochlea and/or the auditory nerves (sensorineural) in the inner ear or a mixture of conductive and sensorineural components.^10,15^

Although the array of techniques used in the diagnosis of sensorineural hearing loss (SNHL, the predominant form of hearing loss worldwide) is progressively being expanded and refined,^14^ developing therapeutics to treat the onset of SNHL is proving to be considerably more difficult to realize^13,14^—thus placing a premium emphasis on prevention.^12,16^ A compelling case for prevention is noise-induced hearing loss (NIHL), one of the most common types of SNHL.^17,18^ Noise pollution is a growing health problem around the world.^19–22^ For example, in the United States alone, it is estimated that more than 25% of the adult population has measurable hearing loss caused by exposure to harmful noise.^10^ Irreversible hearing loss can also be the product of disease^12,16,23,24^ and is often an unfortunate side effect of the aminoglycoside antibiotics^25–30^ and platin-based anticancer drugs.^10,27,28,30–36^ Regrettably, once damage has occurred, hearing aids and cochlear implantation are the only compensatory options presently available for affected individuals.^37^

There is a substantial and growing worldwide unmet medical need for a pharmaceutical approach to treating hearing impairment.^38,39^ SNHL results from damage to the organ of Corti (Fig. 1), causing the degeneration of spiral ganglion neurons due to excessive injury and/or untoward death of cochlear hair cells in the inner ear,^10,40,41^ in most cases caused by internal or external pathologic factors, including infectious or inflammatory processes, ototoxic drugs, noise overstimulation, as well as the normal aging process.^13,16,25,40,42^ Unexpectedly, a recent meta-analysis of numerous epidemiological studies exposed a possible link between presbycusis (also known as age-related hearing loss, ARHL) and cognitive decline, cognitive impairment, and dementia—suggesting that ARHL may be a relevant biomarker and a targetable modifiable risk factor for dementia.^43–46^ However, given that advanced age itself is a leading risk factor for dementia,^1,47–49^ the meta-analysis correlation is not surprising.

## Mitochondrial Dysfunction in Hearing Loss

Mitochondria mitigate cell protection, survival, and function^1,50–52^ (Hoffman ME, Augsburger BN, Foradori CD, et al. Neuroprotective effects of carnitinoid compounds in rodent cellular and in vivo models of mitochondrial complex I dysfunction. 2018; submitted) and, over time, they succumb to an increasing cumulative degradation of their cellular energy production and performance—driving the cell toward death (apoptosis) and/or premature senescence (Hoffman et al., submitted).^40,47,48,53–59^ Energy-demanding cells such as neurons and the vestibulocochlear hair cells (Fig. 1) are especially vulnerable to mitochondrial dysfunction^60,61^ and, consequently, hearing impairment/deafness is a characteristic clinical symptom of several neurodegenerative mitochondrial disease phenotypes.^24,25,44,62–66^

Focusing on the genetic basis of hearing loss, it is worth noting that many mutations in the mitochondrial genome (mitochondrial DNA, mtDNA), as well as in the nuclear genome (nuclear DNA, nDNA), are known to cause hearing deficits (Table 1).^24,62,63,65–67^ Alterations in certain regions of mtDNA associated with deafness are also associated with a host of other disorders, such as myopathy including cardiomyopathy, diabetes, and parkinsonism.^68–72^

**Table 1.** **Representative Mitochondrial Disorders Associated with Hearing Impairment/Deafness**

Disease	Abbreviation	mtDNA/RNA^a^	Defect, presence of symptom, sign or finding, and other notes
Aminoglycoside-induced deafness	AID	Mutation in rRNA	Also associated with Parkinson's disease
Kearns-Sayre syndrome	KSS	Large-scale deletions	Possible presence of sensorineural hearing loss
Mitochondrial encephalomyopathy, lactic acidosis, and stroke-like episodes	MELAS	Mutation in tRNA	
Maternally inherited deafness and diabetes	MIDD	Mutation in RNA	Phenotypically and genotypically heterogeneous
Mohr-Tranebjaerg syndrome	MTS	Mutations in nDNA	Causes defects in mitochondrial protein import machinery
Myoclonic epilepsy with ragged red fibers	MERFF	Mutation in tRNA	
Neuropathy, ataxia, and retinitis pigmentosa	NARP	Mutation in mRNA	Possible presence of sensorineural hearing loss
Progressive external ophthalmoplegia	PEO	Multiple deletions	Ophthalmoplegia is a clinical hallmark of multiple deletions in mtDNA

^a^ Unless otherwise noted.

In addition, mitochondria are key regulators of our innate and adaptive immune responses to viral infections.^50,73–76^ Often overlooked among the many causes of impaired hearing, including deafness, is virus-induced hearing loss.^77–79^ Although the mechanisms of hearing loss/deafness associated with viral infections remain largely undefined,^78,80^ viral infections activate a cascade of mitochondrial antiviral innate immune responses that include nuclear factor kappa B (NF-κB)^50,75,76,80,81^ and nuclear factor erythroid 2-related factor 2 (Nrf2)/antioxidant response element (ARE) signaling pathways.^1,50,76^

Unmitigated oxidative stress (which is primarily caused by mitochondrial dysfunction) and epigenetically altered expression of genes sensing oxidative stress are significant contributors to the pathogenicity of neurodegenerative disorders (Hoffman et al., submitted).^47,52,54,76,82,83^ While a full understanding of these pathways awaits further study, it seems clear that epigenetics plays a significant role. Mammalian vestibulocochlear hair cells are a stress-sensitive, nonregenerative cell type and, like the retinal cells of the eye,^1^ are not replaced when they are injured or die.^10,13,14,29,57,60,82,84–86^ Interestingly, emerging research indicates we selectively amplify directional sound in a noisy environment by unconsciously utilizing eye–ear coordination to integrate visual cues with the auditory information.^87,88^ Assessing vision and oculomotor function is essential in the diagnostic evaluation of vestibulocochlear auditory impairments,^89^ particularly in patients with idiopathic etiology.^90,91^

## α-Lipoic Acid, l-Carnitine, and Butyrate

Impressive advances in gene therapy^8,14,92–94^ and regenerative medicine are making inroads toward regenerating hair cells with the aim of reversing hearing loss.^41,95^ Some of the achievements demonstrate therapeutic potential,^8,86^ but a clinical application is still a long way off.^24,39^ In the more immediate future, shielding hair cells from oxidative damage and/or rescuing injured hair cells from falling into apoptosis by pharmacological treatment with free radical scavenging antioxidant compounds portend a promising therapeutic approach.^13,14,29,30,57,96–99^

**FIG. 2.**
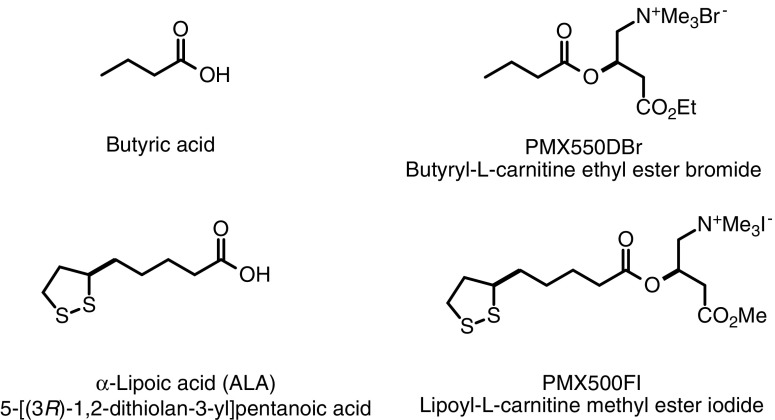
Chemical structures of butyric acid, ALA, and their corresponding carnitine esters. ALA, α-lipoic acid.

Upregulating *Nrf2/ARE* gene expression pathways and/or suppressing NF-κB signaling are cogent targets for pharmaceutical intervention strategies.^34^ Many natural and synthetic compounds are known inhibitors of NF-κB signaling^100^—butyric acid (butyrate)^50,101–105^ and α-lipoic acid (5-[(3*R*)-1,2-dithiolan-3-yl]pentanoic acid)^50,106–110^ (Fig. 2) are among them. Importantly, butyric acid and α-lipoic acid (ALA), as well as their respective corresponding l-carnitine esters PMX550DBr and PMX500FI (Fig. 2), also act as antioxidant histone deacetylase (HDAC) inhibitors (HDACi) (Hoffman et al., submitted).^47,50,54^ noted for effecting favorable epigenetic modulation of the cell survival protein, B cell lymphoma 2 (Bcl-2) with respect to the proapoptotic protein, Bcl-2-associated X protein (BAX) in a BAX/Bcl-2 ratio of 1:2.^54,110–113^ Again, note the implied potential of drugs that modulate epigenetic pathways.

ALA has been extensively researched as a neuroprotectant,^114–119^ acting on signaling mechanisms through both receptor-mediated pathways and nonreceptor-mediated antioxidant processes in a variety of cell types^110,115,116,119,120^—including cochlear hair cells.^31,121,122^ In humans, ALA is a functionally versatile endogenous molecule enzymatically synthesized in mitochondria from octanoic acid.^123^ It is a key cofactor in the construction of vital metabolic multienzyme complexes, including pyruvate dehydrogenase and the glycine cleavage system.^123^ It is also a strong antioxidant^1,119,124,125^ and anti-inflammatory^109,119,125^ agent capable of activating and modulating signal transduction pathways,^109,119,126^ upregulating the expression of nerve growth factor, and augmenting the conduction velocity of motor nerves.^127^

The expression of ∼1% (∼200–250 genes) of the protein-coding human genome is modulated in concert with the Nrf2/ARE signaling pathway.^117,128^ ALA is a potent activator of Nrf2, a transcription factor encoded by *NFE2L2* that helps regulate cellular redox balance and protective antioxidant and phase II detoxification responses in mammals.^50^ Dietary antioxidant supplements are commonly sought by patients and caregivers for treating primary mitochondrial disorders.^23,65^ The role of antioxidants in prevention of age-related hearing loss has been reviewed by Tavanai and Mohammadkhani.^129^ In one of the reviewed studies, C57BL/6 mice fed with control diet or diet containing 1 of 17 antioxidant compounds (acetyl-l-carnitine, *N*-acetyl-l-cysteine (NAC), ALA, carotene, carnosine, coenzyme Q_10_, curcumin, tocopherol, epigallocatechin-3-gallate, gallic acid, lutein, lycopene, melatonin, proanthocyanidin, quercetin, resveratrol, or tannic acid), ARHL was nearly completely prevented by ALA and coenzyme Q_10_ and partially by NAC, but not by the other compounds.^130^ Unfortunately, this strategy showed no significant benefit in an interventional human study.^131^

However, the results from the Polanski and Cruz^131^ study may not truly address the ability of antioxidants to prevent ARHL because the design of the study was not directed toward prevention, and damaged cochlear hair cells are not restored by antioxidants.^129^ In studies aimed at preventing hearing loss in aged animals, ALA was shown to confer significant hearing preservation.^34,108^ Similar results between human and animal studies^99^ were also observed with the use of l-carnitine—an endogenously synthesized molecule mostly obtained from the diet.^65^

NF-κB is a transcription factor that regulates the expression of a variety of genes involved in inflammation and immunity.^81,104,105^ Sodium butyrate is a well-documented HDAC inhibitor^18,27,54,101,105^ that has demonstrated anti-inflammatory NF-κB inhibition properties.^50,101–105^ Butyrate mediates NF-κB activation by rescuing the redox machinery and controlling reactive oxygen species^105^ that are highly injurious to hair cells^18,132^ by suppressing the NF-κB signaling pathways.^105^

Although ALA and butyrate are common food and diet supplements that can be safely taken in high doses, their bioavailability is not prolonged or sustained at an effective therapeutic level.^50^ Furthermore, a recent Phase I clinical trial in age-related macular degeneration evaluating the safety and tolerability of ALA in 15 subjects, 65 years of age or older, showed that high doses (800–1200 mg) of racemic ALA cannot be tolerated very well by patients.^133^ Thus, in the treatment of hearing loss, a need for ALA and butyrate derivatives having more clinically suitable pharmacokinetics is a challenging pharmaceutical objective.

## Concluding Remarks

Hearing impairment is a major global health concern; its massive impact seemingly unrecognized until recently, and the affected population largely untreated. Preventing, or at least delaying or reducing, some hearing loss may be possible by avoiding excessive noise exposure and addressing contributory factors such as cardiovascular risk, infectious diseases, neurological disorders, and drug toxicity. However, these interventions will not be sufficient given the sheer magnitude of the problem. Thus, in view of recent advances in our understanding of the underlying mechanistic pathways—both mitochondrial and epigenetic—that govern hearing function, coupled with new drug discovery paradigms that can today be exploited to identify new and effective therapies, the time is ripe to tackle hearing loss with novel medicines. Alcmaeon of Croton remarked that vision and hearing are constructs of the brain. We see and hear in our dreams and in some aspects of disease conditions, such as high fever, schizophrenia, psychosis, or the later stages of dementia, and our dreams may blend into our conscious state immersed in auditory and/or visual hallucinations and delusions. The most common hallucination in schizophrenia is hearing voices.^134,135^ Finally, readers are directed to Table 2 for a summary of key points related to otologic disorders.

**Table 2.** **Selected Key Points**

**FIG. 3.**
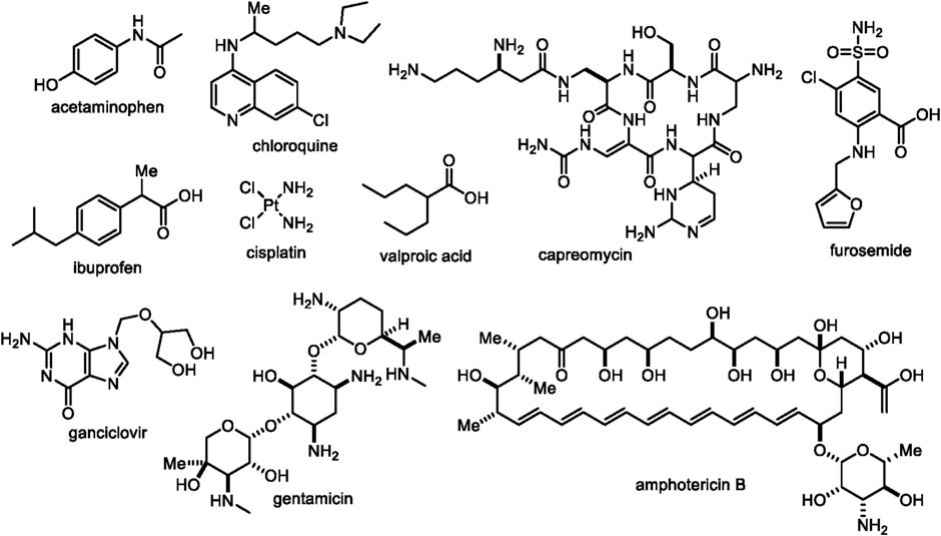
Wide ranging chemical structures of potentially ototoxic drugs.

• Roughly 1 in 15 people worldwide—about 500 million—suffer from disabling hearing loss; two to three times that number have mild-to-complete hearing loss.^12,13^
• Recent studies proclaim hearing problems as the fourth leading cause of YLDs; clearly a major global health concern.^10–12^
• Hearing impairment has been called an “invisible disability” despite its significant consequences, economic and personal; the impact is most substantial in LMICs, where >80% of people with hearing loss reside.^44^
• Noise exposure is a major cause of deafness and hearing impairment (i.e., noise-induced hearing loss); cardiovascular risk caused by diabetes and smoking is also associated with hearing loss.^22^
• Hearing impairment in children and adults may also present as sequelae of cytomegalovirus, Ebola virus, and other serious infections.^14,75,77,121^
• Advanced age is a major risk factor for hearing loss (i.e., presbycusis, age-related hearing loss), with U.S. prevalence nearly 70% over age 70; indeed, age-related hearing loss may prove to be a useful biomarker and treatable risk factor for cognitive decline or impairment, including Alzheimer's disease.^43–46^
• Hearing loss has been observed following TBI, and while it is significant clinically it is yet to be well characterized.^92,136^
• Genetics, both mitochondrial and nuclear, and demographics (educational level, race/ethnicity, sex) have an influence on, or are associated with, hearing disorders.^24,62,63,65,66,72,82^
• The most common congenital sensory impairment is hearing loss, affecting between 1 in 300 to 500 newborns and children to the age of 4; one example results from disruption of a gene that encodes a major component of cochlear gap junctions.^137^
• It is not uncommon to see links between sensorineural deficits in both the ear and the eye; RP, an inherited eye disease, is in some cases associated with reduced hearing ability.^65,66,75,89^
• Sensorineural hearing loss is found in mitochondrial respiratory chain disorders, and mtDNA mutations represent one of the most important causes of hearing loss Table 1^24,62,63,65,66^; given the high energy demands of hearing, mitochondrial involvement should not be a surprise.^60,61^
• Certain drugs (Fig. 3), notably aminoglycoside antibiotics (gentamicin), antivirals (ganciclovir), antifungals (amphotericin B), antimalarials (chloroquine), antituberculosis agents (capreomycin), cardiovascular drugs (furosemide), anticonvulsants (valproic acid), cisplatin (platinol), and immunosuppressants (tacrolimus), can result in significant hearing loss.^16,28^ Ototoxicity is a potential side effect of some commonly used NSAID and related medications, including acetaminophen (paracetamol) and ibuprofen when taken in very high doses or used chronically (≥2 days/week).^42^ In lieu of a recent study suggesting that, if started early enough, a daily regimen of ibuprofen can prevent the onset of Alzheimer's disease,^138^ ototoxicity in this protocol is an important consideration to take into account.
• From a pharmaceutical perspective, the chemical structural diversity (Fig. 3) of potentially ototoxic drugs is noteworthy.
• Drug discovery to identify novel therapeutics that protect hair cells from toxic insults is experimentally challenging owing to the inaccessibility of the inner ear, but zebrafish and other animal models have been explored; screens have identified multiple potential drug classes of interest, for example, antioxidants,^122^ and other compounds acting on classical GPCR neurotransmitter systems (i.e., adrenergic, dopaminergic, serotoninergic) and estrogen receptor modulators.^28,29,32,37,57,65,99^
• Biotechnology and pharmaceutical companies have recognized the unmet medical need and therapeutic potential of new drugs for hearing impairment, as exemplified by ventures such as Aurin, Auris, Autifony, Decibel, Frequency, Novus, Otonomy, Sensorion, and Sound, among others^10,13,31,97^; still relatively untapped in this respect are epigenetic and mitochondrial targets.^8,14,18,26,27,30,34,57,82,98,99,132,139–141^

GPCR, G-protein-coupled receptor; LMICs, low- and middle-income countries; NSAID, nonsteroidal anti-inflammatory drug; RP, retinitis pigmentosa; TBI, traumatic brain injury; YLDs, years lived with disability.

## Abbreviations Used

ALA: α-lipoic acid
ARE: antioxidant response element
ARHL: age-related hearing loss
BAX: Bcl-2-associated X protein
Bcl-2: B cell lymphoma 2
GPCR: G-protein-coupled receptor
HDAC: histone deacetylase
HDACi: histone deacetylase inhibitors
LMICs: low- and middle-income countries
mtDNA: mitochondrial DNA
NAC: N-acetyl-L-cysteine
nDNA: nuclear DNA
NF-κB: nuclear factor kappa B
NIHL: noise-induced hearing loss
Nrf2: nuclear factor erythroid 2-related factor 2
NSAID: nonsteroidal anti-inflammatory drug
RP: retinitis pigmentosa
SNHL: sensorineural hearing loss
TBI: traumatic brain injury
WHO: World Health Organization
YLDs: years lived with disability
